# The American Medical Association

**Published:** 1880-04-20

**Authors:** 


### *THE AMERICAN MEDICAL ASSOCIATION.*

The American Medical Association was appointed to assemble in New York City, June the 1st.

It will probably be one of the largest and most important meetings of the Association ever held since its origin.

We are assured that the Profession of New York will give to the Delegation a reception which will be in full accord with that enlarged and generous hospitality which she has ever maintained. The number and intelligence of the Profession in the city, her colleges, the eminent men connected with them, and the many and varied attractions which she possesses as the great metropolis of the Union, all combine to fit her in an eminent degree as a place for the assembling of the medical talent and wisdom of the Nation.

The Medical Editors' Association will meet on Monday, May 31st, before the meeting of the American Medical Association. The Senior Editor of this Journal, having been honored with the position of President of the Medical Editors' Association, feels in duty bound to be present, and anticipates much pleasure in meeting with his brethren of the quill. He regards the Editors' Convention as a far more important body than many seem to consider it. True it is that in its initial stages its purposes and objects were not well understood, and it has been treated as a social gathering merely, rather than as a business association having great and important responsibilities. If the medical press has not heretofore exercised a great power, it is certainly destined to do so. The rapidly-increasing number of readers, made necessary by the growing educational demand, of the country, and the advances in all departments of medical science, will soon tell wonderfully, not only upon the Profession, but through them upon the masses everywhere. This power is in the hands of the medical editors, and only awaits co-operative efforts and a clear understanding of the proper methods to be used to develop and wield a mighty influence for good. Herein is the great lever for the promotion of professional unity and brotherhood, and for the elevation of the standard of medical education, about which so much has been said of late years. The responsibility for the low state of medical education rests with the medical press not less than with the colleges. Without the aid of the medical press the colleges cannot hope to succeed in their efforts to raise the standard of education.

Medical societies are important auxiliaries, but they require to be stimulated and encouraged by the journals. It is well, therefore, that an Editors' Association has been instituted. Let it be encouraged by all means, and let every editor who can possibly do so go to New York and join the Association; let harmony and good feeling prevail, and let them work together and vie with each other in developing the medical literature of the country, and in elevating the standard of medical education in the American Union.

The Association of Medical Colleges will also convene in New-York, in advance of the sessions of the American Medical Association. About thirty schools have already connected with this body, and others will doubtless join at the next meeting. It is, therefore, a power which is likely to exert a controling influence upon the Profession and its destiny in this country. Its responsibility is great; it should take high ground, but should act with "wisdom, justice and moderation," having due regard to the rights and interests of all, and to the impoverished condition of the Southern section of the Union. These institutions, we doubt not, will be found ready and anxious to unite with their brethren of the Association in an onward and upward movement; but let us march together steadily, cautiously, surely,—not by rash and hasty bound, but step by step until we shall have reached together the empyrean heights.

